# Role of carbohydrate-active enzymes in brown planthopper virulence and adaptability

**DOI:** 10.3389/fpls.2025.1554498

**Published:** 2025-04-04

**Authors:** Fang Liu, Jing Xiao, Xin-Feng Wang, Ya-Xuan Wang, Hou-Hong Yang, Yu-Biao Cai, Feng-Xiang Lai, Qiang Fu, Pin-Jun Wan

**Affiliations:** ^1^ The National Key Laboratory of Rice Biological Breeding, China National Rice Research Institute, Hangzhou, Zhejiang, China; ^2^ Hubei Insect Resources Utilization and Sustainable Pest Management Key Laboratory, College of Plant Science and Technology, Huazhong Agricultural University, Wuhan, Hubei, China

**Keywords:** brown planthopper, CAZymes, plant-insect interaction, rice defense response, rice

## Abstract

**Introduction:**

Herbivorous insects, including the brown planthopper (BPH), Nilaparvata lugens, are among the most damaging pests to agricultural crops worldwide, particularly rice. These insects employ a variety of strategies to overcome plant defenses, including the secretion of carbohydrate-active enzymes (CAZymes) that degrade plant cell walls. While CAZymes are well-studied in other insect species, their role in BPH virulence remains largely unexplored.

**Methods:**

This study aims to address this gap by analyzing CAZymes in 182 insect genomes, followed by a detailed genomic and transcriptomic analysis of BPH.

**Results:**

We identified 644 CAZymes in BPH, including enzymes related to plant cell wall degradation. Through quantitative real-time PCR (RT-qPCR) and subcellular localization experiments, we found that 5 candidate genes exhibited increased expression during feeding on the susceptible rice variety TN1, a well-characterized variety highly susceptible to BPH and these genes were localized to the plasma membrane. Our results suggest that BPH CAZymes play a critical role in the insect's ability to feed and damage rice plants.

**Discussion:**

This study provides valuable insights into the molecular mechanisms underlying insect adaptation and virulence in the co-evolutionary process between plants and herbivorous insects. By exploring the function of pest-related genes in the BPH and examining their differential responses in rice varieties with varying resistance to BPH, we aim to contribute to the development of targeted pest management strategies.

## 1 Introduction

Through the millions of years, the co-evolution between the plants and phytophagous insects brought up a number of different strategies from the insects in taking on nutritional matter from the host plant. Herbivorous insects are estimated to destroy from 10 to 18% of the world’s total annual yield of crops (Oerke, 2005). With the presence of the chewing and pierce-sucking types of mouthparts, herbivorous insects inflict injury within an expansive array of plant parts that ranges from aerial down to the subterranean due to the consumption of main metabolites such as carbohydrates, lipids, and proteins (Felton et al., 1999; Walling, 2000). Plants respond by forming chemical barriers, which include the production of secondary metabolites such as alkaloids and terpenes, phenolic compounds, and the development of physical or mechanical barriers to the insects’ attachment, feeding, and oviposition activities (Salerno et al., 2024).

The plant cell wall (PCW) forms part of the critical physical barriers against plant invasion by pests (Calderón-Cortés et al., 2012; Underwood, 2012). Essentially composed of cellulose, hemicellulose with a network of pectin, the PCW constitutes a physical barrier by improving mechanical strength of plant tissues hence reducing digestibility of food—the first line of defense against herbivore attack (Calderón-Cortés et al., 2012; Santiago et al., 2013). To utilize vegetal and fungal biomass, decomposers need to secrete enzymes hydrolyzing plant and fungal cell wall components like cellulose, hemicellulose, and chitin. CAZymes are listed in a curated database in which the enzymes have been classified into their structural families and carbohydrate-binding modules, enabling degradation of polysaccharides of both plant and fungal cell wall (Drula et al., 2022).

CAZymes play a pivotal role in the breakdown of complex plant polysaccharides, including cellulose, hemicellulose, and pectin, into smaller sugar molecules such as glucose and xylose. These degradation products serve as essential energy and carbon sources for insect metabolism, supporting key physiological processes such as growth, development, and reproduction. The specificity of CAZymes in targeting distinct components of plant cell walls significantly influences the insect’s ability to extract and utilize nutrients, thereby contributing to its overall metabolic fitness and survival (Calderón-Cortés et al., 2012; Fu and Yang, 2023). Other related works described the presence of certain CAZymes in insects, such as termites, playing a major importance in higher-order complexity, including digestion of plant material comprising cellulose and hemicellulose (Beránková et al., 2024; Cleveland Lr et al., 1934; Dar et al., 2022). Transcriptomic analyses of gut microbiomes in herbivorous insects, such as black soldier fly larvae (*Hermetia illucens L*), have also emphasized the central role of CAZymes in the degradation of lignocellulose (Kariuki et al., 2023; Treves and Martin, 1994). Studies of CAZymes in plant pathogenic fungi have shown that they are virulence factors, allowing the pathogens to degrade plant cell walls and penetrate host tissues (Liu et al., 2023a; Liu et al., 2023b; Kubicek et al., 2014; Ma et al., 2019).

Among them, the Hemiptera form one of the five main orders of insects and thus harbor a very broad variety of feeding guilds together with their symbiotic microbes (Bennett and Moran, 2013, 2015; Douglas, 2016; Gupta and Nair, 2020). One important agricultural insect pest, belonging to Hemiptera, is the brown planthopper (BPH): *Nilaparvata lugens*. This poses an enormous risk to rice production in all growing parts of the world (Shen et al., 2023; Sōgawa, 1982). BPH inflicts injury to plants by using stylet mouthparts for probing and penetrating plant cells, sucking out phloem sap that develops into plant death further leading to major economic loss in yield (Backus et al., 2005). BPH serves as a key vector for viral pathogens such as Rice Grassy Stunt Virus (RGSV) and Rice Ragged Stunt Virus (RRSV). While the primary mechanism of virus transmission occurs through insect-mediated phloem feeding (Hao et al., 2008; Claridge and Den Hollander, 1983; Liu et al., 2020; Sōgawa, 1982), CAZymes may contribute indirectly by facilitating viral entry. Studies on plant-pathogenic fungi suggest that CAZymes degrade plant cell walls to weaken structural integrity, thereby promoting pathogen invasion (Munzert and Engelsdorf, 2025). Similarly, certain CAZymes secreted by phloem-feeding insects may contribute to cell wall loosening and increased permeability, potentially enhancing viral transmission efficiency. Further studies are needed to determine whether specific BPH-derived CAZymes play a direct role in facilitating viral entry into host tissues. Exploring the precise role of these enzymes in plant damage caused by BPH not only helps uncover the molecular basis of pest feeding behavior but also provides essential insights into the specific mechanisms of CAZymes in BPH infestation.

In this study, we aimed to investigate the abundance and functional role of CAZymes in BPH. We conducted a comparative analysis of CAZymes across 182 insect genomes, revealing significant differences in CAZyme profiles between holometabolous and hemimetabolous insects, as well as between polyphagous and oligophagous species. We focused specifically on the CAZyme transcriptome of BPH, which contains a high proportion of CAZymes (3.23%). Bioinformatic analysis identified 644 putative CAZymes in the BPH genome, with 256 (39%) belonging to glycoside hydrolase families implicated in plant cell wall degradation (e.g., GH9, GH45). Functional annotations were derived from homology to biochemically characterized CAZymes in the CAZy database. While experimental validation in BPH is pending, these predictions are consistent with enzymatic activities reported in closely related hemipterans (Fu and Yang, 2023). Our findings suggest that these CAZymes play a crucial role in BPH’s virulence and provide new insights into the molecular mechanisms underlying its adaptability and functional diversification. These results offer potential avenues for developing targeted pest management strategies.

## 2 Materials and methods

### 2.1 Insect materials

TN1-BPH, a population of brown planthoppers reared on TN1 rice was used in this study. The TN1-BPH colony was initially collected from rice fields in Hangzhou, China, and has been maintained on Taichun Native 1 (TN1) rice in a climate-controlled chamber (26 ± 1°C, 85 ± 5% RH) for over 12 years at the China National Rice Research Institute (CNRRI). All adult insects were transferred to new culture dishes 24 hours after emergence and subjected to a 4-hour starvation period before being introduced to the rice plants. This pre-conditioning protocol ensures consistent experimental conditions and minimizes variability in insect behavior.

### 2.2 Plant materials

Six indica rice varieties were used in this study, including one susceptible variety, TN1, and five resistant varieties carrying different BPH-resistance genes: Mudgo (*Bbp1*), IR42 (*bph2*), IR56, RHT (*Bph3*), and Babawee (*bph4*). All pre-germinated seeds were sown in pots (17 cm in diameter) filled with multi-purpose compost and cultivated in a net house. The experiments were conducted under controlled natural light conditions, with an average photosynthetically active radiation (PAR) intensity of approximately 1200-1500 μmol·m^-2^·s^-1^, as measured using a quantum sensor. To ensure consistency, the net house was equipped with shade nets to mitigate excessive light intensity during peak hours, and temperature and humidity were monitored continuously using data loggers. The temperature was maintained between 25-28°C, and relative humidity was kept at 70-80% throughout the experiment. Two weeks later, the plants were transferred to a temperature-controlled greenhouse (26 ± 2°C, 80 ± 5% RH), where they were maintained for an additional 7 days before being used in the experiments.

### 2.3 Bioinformatics analysis

Insect reference genomes were retrieved from Ensembl (https://metazoa.ensembl.org) and OrthoDB (https://www.orthodb.org) using the accession numbers provided in **Supplementary Table S1**. Gene analysis for CAZymes and the prediction of CAZyme gene clusters (CGCs) were performed across the insect genomes. CGCs were identified based on the presence of at least one signature gene encoding sugar transporters, signal transduction proteins, or transcription factors, using dbCAN2 (Zhang et al., 2018). Target substrate predictions for identified CGCs were made using dbCAN2, based on the dbCAN-PUL database and CAZyme subfamilies, employing eCAMI tools. Potential functional domains in proteins from fully sequenced genomes were identified with the InterProScan v5 software package, which searches protein sequences against various signatures from databases such as PROSITE, PFAM, PRINTS, SMART, and TIGRFAMs (Yin et al., 2012). Signal peptides were predicted using SignalP 5.0 program (Almagro Armenteros et al., 2019). We performed pathway enrichment analysis on the genes of interest, including enrichment (Fisher’s exact test was used) in Gene Ontology (GO) using the Cytoscape software platform (version 3.4) (Shannon et al., 2003). The analysis utilized the latest version of the Gene Ontology (GO) database to ensure up-to-date annotations. A threshold of false discovery rate ≤ 0.05 was used to determine significant GO terms.

The phylogenetic gene tree was constructed by extracting the sequences of the longest catalytic domain (>120 amino acids) for each gene. Multiple sequence alignments were performed using MEGA 6 software with default parameters (Tamura et al., 2013). Neighbor-Joining (NJ) trees were constructed using the *p*-distance method with 2,000 bootstrap replicates. The active center of the catalytic domain for each family was derived from the sequence alignment, as described in previous studies (Liu et al., 2018). Additionally, the sequence logo was generated using WebLogo (http://weblogo.berkeley.edu/).

### 2.4 Quantitative real-time PCR

Thirty fifth-instar TN1-BPH nymphs were separately infested on both susceptible and resistant rice varieties. TN1-BPH samples were collected after 6 hours and promptly frozen in liquid nitrogen. Total RNA was extracted from the BPHs and reverse transcribed into cDNA, which served as the template. Specific primers listed in **Table 1** were selected. qRT-PCR analysis was performed with SYBR Green master mix (Takara) in the CFX96TM Real-Time PCR Detection System (Bio-Rad, Hercules, CA, United States). For each PCR reaction, a 20 μL reaction mixture was used with 1 μL cDNA, 1 μL of each primer (10 μM), 7 μL ultrapure water, and 10 μL SYBR buffer. The reaction conditions consisted of a preincubation at 95°C for 2 minutes, followed by 39 cycles of denaturation at 95°C for 5 seconds, annealing at 59°C for 30 seconds, and melting curve analysis from 65°C to 95°C. The melting curve analysis was conducted over a temperature range of 65°C to 95°C. The data were interpreted by confirming the presence of a single peak in the melting curve, which indicates primer specificity and the absence of non-specific amplification products. Amplification of 18S RNA served as the internal control. Relative gene expression was analyzed using the 2^−ΔΔCT^ method.

**Table 1:** Enzyme activity and signal peptide characteristics of CAZymes candidate genes.

Gene ID	Annotation	Signal	CAZy family
MSTRG.7223.4	chitinases	YES	GH20
rna27461		YES	CBM14
MSTRG.15.4		NO	GT24
rna38279	exoglucanase	YES	GH27
rna36994		YES	CBM14
MSTRG.5824.1		NO	GH31
MSTRG.20826.10		NO	GH13
MSTRG.36184.4		YES	GT31
MSTRG.25023.4	acetylglucosamine transferase	NO	GT49
MSTRG.36466.1		NO	GT64
MSTRG.41825.4		NO	GT20
rna25783	β-glucuronidase	NO	GH38

### 2.5 Subcellular localization in rice protoplasts

As previously described, the rice protoplasts were prepared and transfected (Zhang et al., 2011). For a short overview, fragments of 0.5 mm size from the seedlings of rice grown in Hoagland medium at ten days of growth were cut, transferred into 0.6 M mannitol solution, and kept in the dark for 30 minutes. The protoplasts lysed the tissue, which was then transferred into a solution containing 1.5% w/v cellulase R-10, 0.75% w/v macrozyme R-10, 0.5 M mannitol, 10 mM MES at pH 5.7, 0.1% v/v BSA, 10 mM CaCl_2_, and 5 mM β-mercaptoethanol for cell wall degradation. Rice protoplasts were transformed with nuc-tagged bZIP63 and a recombinant vector by polyethylene glycol-mediated transfection. Then, they were incubated in the dark for 16 hours and their green and red fluorescence was monitored using a Zeiss LSM CLSM 980 (Carl Zeiss AG). The imaging was performed using a Zeiss LSM 980 Confocal Laser Scanning Microscope (CLSM) with a 63x oil immersion objective (NA = 1.4). For green fluorescent protein (GFP) imaging, the excitation wavelength was set to 488 nm, and the emission was detected between 500-550 nm. For red fluorescent protein (RFP) imaging, the excitation wavelength was set to 561 nm, with emission detected between 570-620 nm.

### 2.6 Data analyses

Statistical analyses were conducted using R version 4.3.2 within RStudio. To analyze CAZyme proportions among insect species, the proportion of CAZymes was calculated as the ratio of total CAZyme counts to total gene counts for each species. A generalized linear model (GLM) with a binomial distribution and log link function was constructed using the *glm* function in R to evaluate the effects of metamorphosis type and feeding type on CAZyme proportions. This approach enabled the assessment of potential interactions between metamorphosis types and feeding strategies. For gene expression analysis, one-way ANOVA was performed to compare expression levels across different plant treatments, as well as spatial and temporal data. Data normality was assessed using the Shapiro-Wilk test (α = 0.05), and homogeneity of variance was verified with Levene’s test (α = 0.05). For datasets violating parametric assumptions, non-parametric alternatives, such as the Kruskal-Wallis test, were employed. Data were presented as mean ± SE, and normality and homogeneity were checked prior to statistical analysis. Tukey’s HSD *post-hoc* test was used to identify significant pairwise differences. These analyses were conducted using base R function, the *Anova* function from the car package (v3.1-2) and the *tukeyTest* function from the PMCMRplus package (v1.9.10).

## 3 Result

### 3.1 Genomic distribution and abundance of CAZymes in herbivorous insects

**Figure 1:**
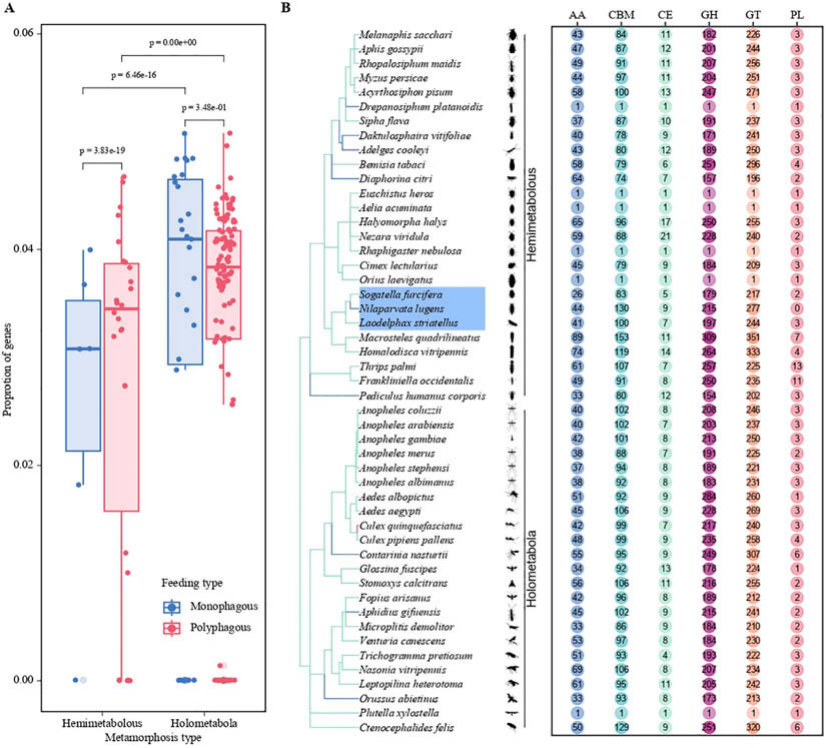
Statistical analysis of the number of CAZymes in herbivorous insects based on genomic data. **(A)** Bar plots depict the proportion of genes in different metamorphosis types (Hemimetabolous and Holometabola) and feeding strategies (Monophagous and Polyphagous). The two colors represent the feeding strategies: blue for monophagous insects and red for polyphagous insects. Statistical significance is indicated by p-values. **(B)** Distribution of CAZyme gene classifications (AA, CBM, CE, GH, GT, PL) among various insect species. The chart illustrates differences in the abundance of CAZyme gene families, emphasizing variation across species.

We investigated the distribution of CAZymes across 182 insect genomes and their abundance. Polyphagous insects have a higher number of CAZymes than oligophagous insects, and holometabolous insects have a higher number than hemimetabolous insects. Our results indicate that the quantity of CAZymes is relatively high in *Schistocerca piceifrons*, *Rhagoletis zephyria*, *Schistocerca americana*, *Bradysia coprophila*, and *Teleopsis dalmanni*, particularly reaching 1018 (about 1.05% of total gene number) in *Schistocerca piceifrons*. In contrast, species with the lowest CAZyme counts (including *Bombus impatiens*, *Bombus terrestris*, *Osmia bicornis*, *Vespa crabro*, and *Vespa velutina*) present fewer than 10 CAZymes, showing very small proportions (less than 0.05%) compared to their total number of genes. Among planthopper species, *Nilaparvata lugens*, *Sogatella furcifera* (White-backed planthopper), and *Laodelphax striatellus* (Small [lanthopper) harbor 675, 592, and 512 CAZymes, corresponding to 3.23%, 3.38%, and 3.31% of their total gene numbers, respectively (**Figures 1A, B**). These proportions highlight the high prevalence of CAZymes, reflecting the adaptive enzymatic capacities of these planthoppers.

### 3.2 CAZyme gene family classification and functional annotation analysis in the brown planthopper

**Figure 2:**
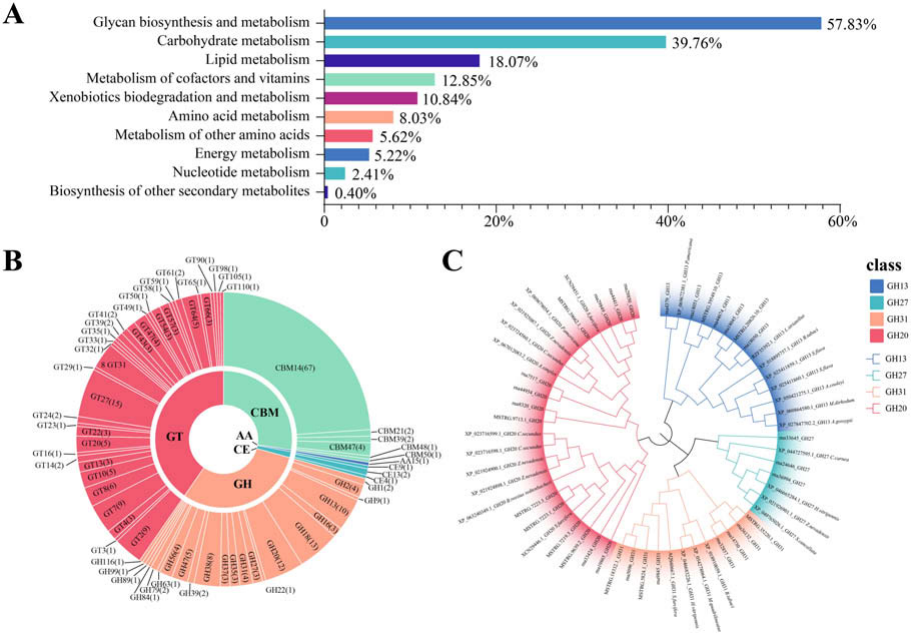
Functional categorization, classification, and phylogenetic analysis of CAZymes in herbivorous insects. **(A)** Functional classification of CAZyme-related genes based on metabolic processes. Bar chart shows the proportion of genes associated with various metabolic categories, including proteolysis (57.83%), carbohydrate metabolism (39.76%), lipid metabolism (18.07%), and others. **(B)** Classification of CAZymes into six major families: Glycoside Hydrolases (GH), Glycosyltransferases (GT), Carbohydrate Esterases (CE), Polysaccharide Lyases (PL), Auxiliary Activities (AA), and Carbohydrate-Binding Modules (CBM). The circular layout illustrates the distribution of CAZyme gene families, with detailed subfamily annotations. **(C)** A maximum likelihood tree was constructed using protein sequences from the glycoside hydrolase (GH) family identified in the brown planthopper, for the plant cell wall-degrading enzymes.

Based on the analysis of CAZymes in the BPH genome, a total of 644 genes were identified, generating 1,675 transcripts. This diverse array encompasses a spectrum of enzymatic functions, including 215 glycoside hydrolases (GH), 277 glycosyltransferases (GT), 130 carbohydrate-binding modules (CBM), 44 auxiliary activities (AA), and a modest 9 carbohydrate esterases (CE), distributed across five distinct gene families. Notably, the polysaccharide lyases (PLs) gene family, which is typically quite prominent in such studies, was conspicuously absent in our findings within the brown planthopper (**Figure 1B**). Given the low abundance of PLs in insects, our subsequent focus will be on the comparison of subfamilies with higher abundance. This may be related to its monophagous nature, as it can only feed and reproduce on rice and common wild rice. The BPH pierces the leaf sheath tissue of rice to ingest phloem sap, which lacks pectin to a large extent. Since pectin is a common substrate for PLs, the lack of pectin in the phloem sap reduces the selective pressure for BPH to retain PLs. Instead, BPH relies on enzymes such as endo-β-1,4-glucanase (GH9 family) and invertase, which are more suited to process phloem-specific components. This is consistent with previous studies that demonstrate the role of these enzymes in the degradation of plant cell wall components in other phloem-feeding insects (Ji et al., 2017). Alternatively, it is possible that BPH has evolved alternative mechanisms to substitute for the function of PLs. For example, a recent study showed that BPH can inhibit the acidification of rice cells during feeding by secreting a salivary carbonic anhydrase (NICA), thereby suppressing the plant’s defense response (Jiang et al., 2024). Plant cell wall degrading enzymes (PCWDEs) from insects have recently been identified as a multigene family of proteins, primarily composed of glycoside hydrolases (GHs) and carbohydrate esterases (CEs). These enzymes play essential roles in the degradation of the cellulose, hemicellulose, and pectin network in the invaded host plant. Herbivorous insects produce an impressive array of PCWDEs to break down the plant cell wall and consume its nutrients, which are vital for their development. For BPH, these enzymes enable the insect to effectively exploit the plant’s resources by degrading key structural components of the host plant cell wall. Diving deeper into the GO analysis, particularly within the Biological Process category, we classified a total of 644 proteins, with 256 (39%) of these associated with carbohydrate metabolism, particularly PCWDEs. Proteolysis proteins accounted for the largest proportion, with 372 proteins (57.8%) (**Figure 2A**). Notably, 282 CAZymes were identified as potentially secreted proteins based on the presence of signal peptides (as detailed in **Supplementary Table S1**). These secreted molecules are likely delivered through the insect’s salivary glands into the host plant, where they may play crucial roles in degrading plant cell walls, suppressing plant immune responses, and facilitating successful feeding.

### 3.3 Classification of carbohydrate−active enzymes genes and phylogenetic tree in BPH

In subsequent analyses, we focused on a subset of enzymes predicted to interact with host constituents, including extracellular matrix polysaccharides and endogenous insect chitin, as classified by the CAZy database. The BPH genome revealed a CAZyme secretome rich in chitinases (GH18, GH20, and GH22) and chitin-active enzymes (CMB14, CMB50, and CE9). Additionally, enzymes involved in the degradation of plant and fungal cell walls were identified, including β-glucanases (GH2, GH16, and GH27), endoglucanases (GH9, GH38, and GH47), and β-glucosidases (GH1), all implicated in cellulose digestion. Pectinolytic enzymes (GH35 and CE13) and amylose-digesting enzymes such as alpha-amylases and alpha-glucosidases (GH13 and GH31) were also present (**Figure 2B**).

A total of 38 glycosyltransferase (GT) families were identified, with GT2 being the most abundant, containing 115 enzymes. Notably, cell wall–degrading enzymes targeting arabinogalactan, xylan, pectin, and mannosyltransferase (e.g., GT31, GT2, GT8, GT47, GT61, GT22, GT23, and GT24) were predicted to interact with GT domain–containing proteins. As GTs directed to the secretory pathway are typically endoplasmic reticulum or Golgi apparatus–resident proteins, they are not further discussed here. Beyond GTs, oxidoreductases such as lytic chitin monooxygenases (AA15) were identified, reflecting their role in chitin modification. AA15 enzymes play a key role in degrading chitin and cellulose through C1 oxidation, which is essential for various physiological activities in insects. These enzymes, often acquired from gut microbiota, assist in the remodeling of the insect exoskeleton during metamorphosis. Over time, AA15 enzymes have evolved the ability to also degrade cellulose, contributing to the insect’s ability to utilize plant material more effectively. In plant-insect interactions, AA15 family proteins hydrolyze key plant cell wall components such as cellulose and chitin, which are integral to the structural integrity of the plant. This enzymatic activity allows insects to break down plant tissues like leaves, stems, and roots, facilitating their feeding and colonization of host plants.

The genome also encoded predicted proteins containing carbohydrate-binding modules, contrasting with prokaryotic and many fungal genomes from biomass-degrading environments. The chitin-binding CBM14 family was associated with CE4 catalytic domains, while the β-glucan-binding CBM39 family was linked to GH16 β-glucanase domains. Additional CBMs capable of binding glucan and starch, including CBM48 and CBM21, were also identified (**Figure 2B**).

The secreted CAZymes encoded by the BPH genome enable the degradation of fungal and plant cell walls, targeting cellulose, xylans, glucans, pectin, and chitin (**Figure 2C**). Among the identified CAZymes, twelve genes, including abundant cellulases, chitinases, and glycosyltransferases, were selected for further study (**Table 1**). Phylogenetic analysis of CAZyme genes revealed that GH20 family genes, which exhibit chitinase activity, are highly similar to the β-N-acetylhexosaminidases found in *Sogatella furcifera* and *Zootermopsis nevadensis*, enzymes involved in chitin hydrolysis. Notably, GH20 sequences in planthoppers form a distinct evolutionary branch compared to other sap-sucking insects, indicating high conservation. Interestingly, the GH20 gene MSTRG.7223.4 shares high sequence similarity with the chitinase XP_063240349.1 from *Bacillus rossius redtenbacheri*, suggesting potential horizontal gene transfer (**Figure 2C**). The possibility of horizontal gene transfer (HGT) involving GH20 genes in BPH is an intriguing hypothesis. While phylogenetic analysis and synteny data suggest potential evolutionary links, further molecular evidence is needed to conclusively demonstrate HGT events. Additional studies, such as comparative genomic analysis across insect species, would provide stronger support for this hypothesis (Intra et al., 2008; Naumov and Stepushchenko, 2011; Shufran et al., 2000). Analysis of other CAZyme families revealed that rna36994 in the GH27 gene family is highly similar to the alpha-N-acetylgalactosaminidase from *Chrysoperla carnea*, while GH13 and GH31 gene family members were located in the genomes of the white-backed planthopper and the tobacco whitefly, respectively (**Figure 2C**). The CBM14 gene is widely distributed among Hemiptera insects and shows significant homology with the genes of *Pristhesancus plagipennis*, *Nesidiocoris tenuis*, and *Popillia japonica* (**Supplementary Figure S1**). Within the GT family, genes such as GT31, GT64, and GT20 were widely distributed among Hemiptera insects, with significant homology to genes found in termites. Highly similar GT family genes were also present in sap-sucking insects, including *Halyomorpha halys*, *Cryptotermes secundus*, and *Homalodisca vitripennis* (**Supplementary Figure S2**). In contrast, GH38 sequences were limited in the BPH genome, and no highly similar sequences were found in other insects, potentially reflecting evolutionary divergence or functional specialization in BPH.

### 3.4 Expression profiles of CAZymes in the brown planthopper in response to host plant changes and developmental stages

We focused on genes from the CAZyme families (GT, GH, and CBM) identified in our enzyme activity analysis (Section 3.3). These families were the most abundant among the 256 enzymes involved in cell wall degradation. These selected genes were those most relevant to BPH’s ability to break down plant cell walls. For example, genes from the GH family (GH20, GH27, GH31, GH13, and GH38) were selected, as these enzymes are involved in the breakdown of hemicellulose and cellulose, which are critical components of the plant cell wall. Additionally, candidate genes from the GT and CBM families were chosen due to their roles in glycosylation and carbohydrate-binding, both of which are essential processes in the degradation of the cellulose/hemicellulose/pectin network in the plant host. To elucidate the expression patterns of CAZYme-encoding genes during BPH feeding, qRT-PCR was performed on 13 candidate genes. The primers used are listed in **Table 2** and include four exoglucanase genes (*rna36994*, *MSTRG.5824.1*, *MSTRG.20826.10*, and *MSTRG.36184.4*), one chitinase endonuclease gene (*MSTRG.7223.4*), three chitinase genes, three glycosyltransferase genes (*MSTRG.15.4*, *rna27461*, and *rna38279*), and one β-glucosidase gene (*rna25783*).

**Table 2:** Gene-specific primers used for quantitative real-time polymerase chain reaction (qRT-PCR).

Gene ID	Forward primer (5′–3′)	Reverse primer (5′–3′)
MSTRG.15.4	TTGGGTTGTGTCTGACTTGTC	AACGGTGCTCCTCCACTTTGC
MSTRG.7223.4	CAGAGCCGGGCCTACTCTTCC	ACCCAGCCTCCAAATCCACAA
rna27461	CCAGTCGCTACCAGTGCTCGC	CTGTCTCCGATCTTGAAGACG
rna38279	CGTCAATGTAGAGTGTGGAGA	GTGAGGGAATGTGGGATGAGG
rna36994	TATTTGGCGTGTCAGAAGGGC	GAATCGTTTGGGGTCAGGCTG
MSTRG.5824.1	CCCTATTTGCCACGAGCAGA	TGTTGAAAGCTGTTTCGCCG
MSTRG.20826.10	GCTCAGAAACGAGGGGATGT	CAACCAAGGTTTGAGGCACG
MSTRG.36184.4	ATGAACGTGTGAGGTTCCCC	GCATCTCGTACACCTGGCTA
MSTRG.25023.4	ATATCAGTACAGGCCGCTGG	CTGAAGTTGCCGAGGTCGAT
MSTRG.36466.1	GTCCAAGTGGACCAACGACT	CGTTCCACGGTGACCTCAAA
MSTRG.41825.4	GCCTGATGTGAACATCGCCA	TCTCAAGTGAGCCGGTGTCTC
rna25783	CTTGGATCGCCTGTGGTAGG	TGGTGCAAGCGCTGAAATTG

**Figure 3:**
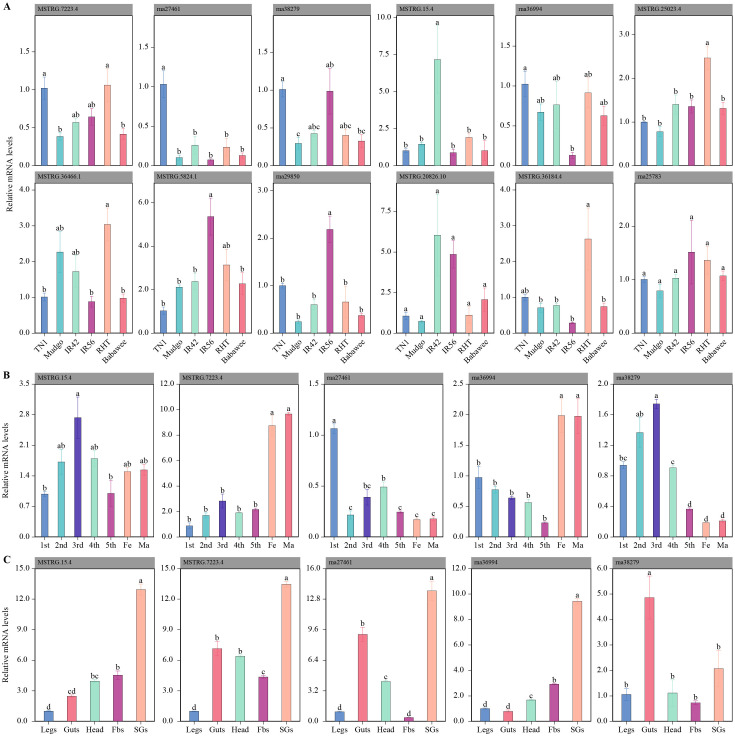
Expression patterns of CAZyme-related genes in the brown planthopper (BPH) under different conditions. **(A)** Expression of CAZyme genes in BPH feeding on various rice varieties with different resistance traits. Bar plots show the relative mRNA expression levels of 12 selected genes across six rice varieties (TN1, Mudgo, IR42, IR56, RHT, and Babawee). Significant differences in expression levels between varieties are indicated by different letters (e.g., a, b, c). **(B)** Developmental stage-specific expression patterns of five CAZyme genes in BPH. Relative mRNA levels are shown for different nymphal instars (1st–5th) and adult stages (female and male). Statistical differences in expression levels are denoted by different letters. **(C)** Tissue-specific expression profiles of the same five genes in various body parts of BPH (legs, guts, head, fat bodies [Fbs], and salivary glands [SGs]). Bar plots illustrate the relative mRNA expression, with significant differences across tissues indicated by distinct letters.

After TN1-BPH fed on five rice varieties with varying BPH-resistance levels, CAZyme genes exhibited distinct response patterns. Compared to TN1 rice, the relative expression levels of four genes (*MSTRG.7223.4*, *rna27461*, *rna36994*, and *rna38279*) were significantly reduced in Mudgo rice, while other genes showed no significant changes. In IR42 rice, *MSTRG.15.4* was significantly upregulated, whereas the expression of other genes remained unchanged. In IR56 rice, two genes (*rna27461* and *rna36994*) were significantly downregulated, while two others (*MSTRG.5824.1* and *rna29850*) were significantly upregulated. Similarly, in RHT rice, one gene (*rna27461*) was significantly downregulated, while two genes (*MSTRG.25023.4* and *MSTRG.36466.1*) were significantly upregulated. In Babawee rice, three genes (*MSTRG.7223.4*, *rna36994*, and *rna38279*) were significantly downregulated, while the remaining genes exhibited no significant changes. Overall, three genes (*MSTRG.7223.4*, *rna27461*, and *rna38279*) were consistently downregulated across multiple rice varieties, whereas three others (*MSTRG.5824.1*, *MSTRG.25023.4*, and *MSTRG.36466.1*) were consistently upregulated. Two genes (*MSTRG.36184.4* and *rna25783*) showed no significant changes (**Figure 3A**).

Through the expression profile analysis of BPH, we identified several genes that were differentially expressed when feeding on various rice varieties. The changes in gene expression reflect the dynamic adaptation of BPH to different host plant environments and highlight the molecular strategies it employs to optimize feeding and survival.

The expression of genes involved in plant cell wall degradation, such as those in the GH and CBM families, was significantly upregulated in BPH feeding on susceptible rice varieties. This suggests that BPH responds to the presence of tougher plant cell walls by activating enzymes capable of breaking down key structural components such as cellulose and hemicellulose. This upregulation of PCWDEs is a key part of BPH’s feeding strategy, enabling it to overcome the physical barriers of rice cell walls and gain access to the nutrient-rich phloem sap.

Conversely, in resistant rice varieties, BPH exhibited a more regulated expression of these PCWDEs, suggesting that the plant’s defenses, including physical barriers and chemical signals, may limit BPH’s ability to break down the cell wall. This is consistent with findings from previous studies where resistant plants induce an increase in physical and chemical defenses that inhibit the activity of herbivores (Plant Defense Against Herbivores: Chemical Aspects) (Tong et al., 2012; Yang et al., 2012; He et al., 2020; Zeng et al., 2021). Additionally, the expression of proteolytic enzymes was higher in BPH feeding on resistant varieties, indicating that BPH may increase the breakdown of proteinaceous components to compensate for any reduced nutritional intake from compromised phloem sap.

Furthermore, BPH’s ability to modulate the expression of oxidoreductases, like AA15 enzymes, suggests that these enzymes play a critical role in both breaking down plant cell walls and in protecting the insect from host-induced oxidative stress. The increased expression of oxidoreductases in response to feeding on resistant rice varieties may also be a mechanism for detoxifying plant defense compounds, allowing BPH to overcome chemical defenses in the plant.

In summary, BPH demonstrates a highly adaptable response to different rice varieties, modulating its gene expression to enhance feeding efficiency and overcome host plant defenses. These expression profile changes are critical to its survival and suggest that BPH has evolved specialized mechanisms to feed on a wide variety of rice plants, from resistant to susceptible varieties, by regulating enzyme production and stress response pathways.

To gain insights into the functional roles of these genes, their tissue-specific expression patterns were analyzed. Secretory proteins, known to play critical roles in feeding, were expected to be overexpressed in the salivary glands or gut tissues compared to other body parts. Based on this hypothesis, qRT-PCR analysis of five candidate genes revealed that four genes (*MSTRG.15.4*, *MSTRG.7223.4*, *rna27461*, and *rna38279*) were highly expressed in the salivary glands, while one exoglucanase gene (*rna36994*) was highly expressed in the midgut (**Figures 3B, C**).

The expression patterns of these five candidate genes were also examined across different growth stages (1st–5th instar, female adults, and male adults). The expression levels of three genes (*MSTRG.15.4*, *rna27461*, and *rna38279*) increased during nymphal development, peaking at the 3rd instar stage. Additionally, two genes (*MSTRG.7223.4* and *rna36994*) exhibited significantly higher expression in the adult stage. These findings suggest that secreted proteins, particularly those expressed in salivary glands or the intestine and responsive to host plant changes, may play essential roles in rice cell wall degradation by BPH.

### 3.5 Subcellular localization of candidate genes in rice protoplasts

**Figure 4:**
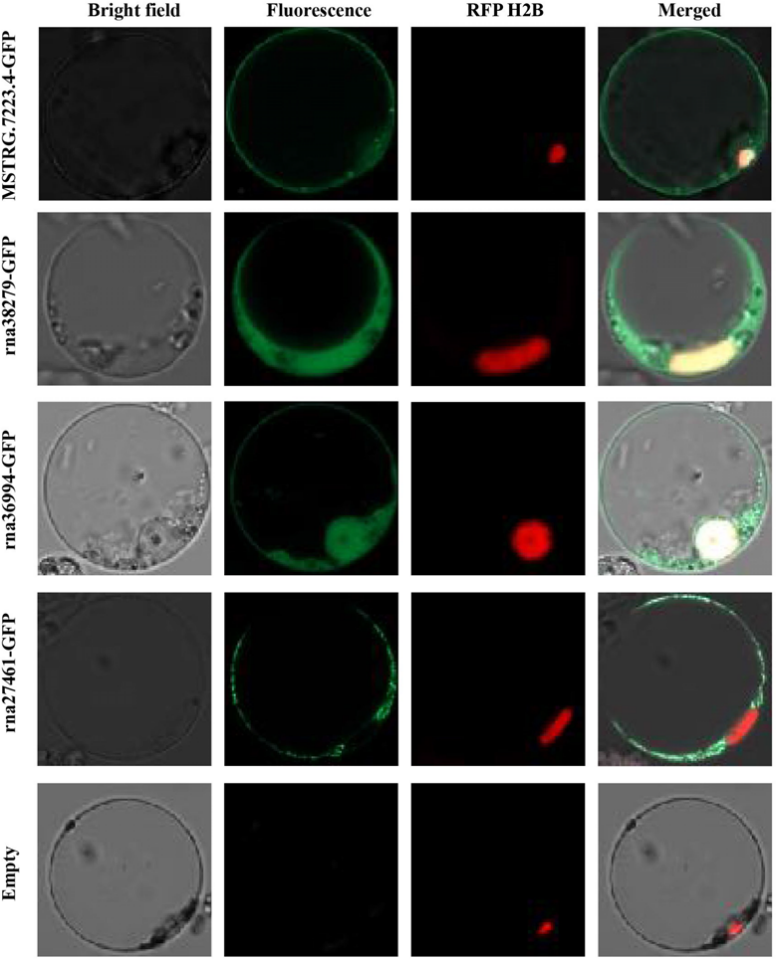
Subcellular localization of CAZyme-related gene products in rice protoplasts. The plot shows the subcellular localization of four CAZyme-related proteins (MSTRG.7223.4, rna38279, rna36994, and rna27461) fused with GFP, observed in rice protoplasts. The empty vector control (Empty) was included as a negative control.

To investigate the subcellular localization of differentially expressed genes in plant cells, we removed the signal peptide from each differentially expressed transcript protein and fused it with GFP for localization tracking (**Table 3**). Rice protoplasts were prepared and transiently co-transfected with nuclear markers to determine whether the GFP signal overlapped with the nuclear localization signal (**Figure 4**). The results showed that three gene-encoded proteins (MSTRG.7223.4, RNA36994-GFP, and RNA38279) localized to both the plasma membrane and the nucleus, whereas one protein (RNA27461) localized exclusively to the plasma membrane. These findings indicate that most of these genes are localized in the cytoplasm and nucleus, suggesting potential roles in cellular signaling or interactions with host cellular components (**Figure 4**).

**Table 3:** Gene-specific primers used for subcellular localization.

Vector name	Forward primer (5′–3′)	Reverse primer (5′–3′)
pAN580-rna27461	F-ATGGCTCGAACGACGTTAGTGGTA	R-GTTGGCGTCGTCATAGAGGTCCT
pAN580-rna36994	F-ATGCAGCACAAAGATGTGTT	R-GGTCGTATGTTTTTGGATCAGCAT
pAN580-MSTRG.7223.4	F-ATGTTGGAGTTGAACCGGATACAG	R-ATTCCTGTTCAAACTCCTCAGGTC
pAN580-rna38279	F-ATGAGGTCAAACAGTCAATCAGC	R-TGACTTTTTCGCCTTCTTGTCCT

F and R are the homologous arms of the subcellular localization PNA580-GFPF vector, and the base sequences of F and R are as follows: F: GGACAGCCCAGATCAACTAGT R: GTCCTCGAGACGTCTCTA.

## 4 Discussion

### 4.1 The richness of CAZyme genes in phytophagous insects is related to their adaptability

Populations exhibiting high genetic diversity typically demonstrate enhanced adaptability, allowing them to better withstand environmental changes and stresses (Soliveres et al., 2016). The brown planthopper has become the most significant pest of rice due to its strong adaptability (Hogenhout and Bos, 2011). Our analysis revealed that BPH possesses 675 CAZymes, which represent approximately 3.23% of its total gene repertoire. While there is growing evidence that primary herbivores, including phloem-feeders, can modify plant defense mechanisms and influence plant vulnerability, direct experimental evidence connecting CAZyme diversity in primary herbivores to increased susceptibility to secondary herbivores is currently limited. It remains to be further demonstrated whether primary herbivorous insects, including phloem feeders, can alter plant defense mechanisms and affect plant vulnerability (Moreira et al., 2018; Thaler et al., 2012; Fernández de Bobadilla et al., 2021).

The diversity and functionality of carbohydrate-active enzymes in insects are intricately linked to their dietary habits, developmental stages, and host interactions. CAZymes play a critical role in dietary adaptation. For instance, herbivorous insects like the rice planthopper (BPH) possess a diverse array of CAZymes involved in the degradation of cellulose and hemicellulose, which are essential for digesting plant cell walls and facilitating insect feeding (Will et al., 2007). Similarly, wood-feeding beetles from the Scolytinae subfamily exhibit CAZymes that enable adaptation to various wood types (Willis et al., 2017).

In holometabolous insects like *Drosophila melanogaster*, enzyme profiles vary between larval and adult stages, with larvae requiring enzymes to digest fruits, while adults have reduced enzymatic needs (Kariuki et al., 2023). In contrast, hemimetabolous insects such as BPH maintain consistent CAZyme profiles throughout life stages, enabling them to feed on similar hosts like rice plants (Michalik et al., 2018). Host specificity also influences CAZyme diversity; monophagous insects like *Bemisia tabaci* possess specialized CAZymes for their host’s carbohydrate profiles, while polyphagous insects like locusts have a broader array of CAZymes, aiding their ability to process various plant materials (Trinca et al., 2023).

The broad host range and both herbivorous and carnivorous feeding habits in insects like *Schistocerca piceifrons* and *Rhagoletis zephyria* further suggest the presence of various digestive enzymes (Fu and Yang, 2023). Non-obligate parasites, like certain pathogens, require extracellular enzymes to kill host cells and use dead tissue for nutrition, while obligate parasites maintain host viability (Liu et al., 2023a; Richardson et al., 2011). Similarly, oligophagous insects evolve potent CAZymes to adapt to specific host plants while avoiding plant immune responses (Aluja et al., 2020; Ogata et al., 2023; Salerno et al., 2024).

An intriguing question raised by our findings is whether BPH’s CAZymes could be applied as exogenous elicitors to stimulate plant immune defenses. Similar to the way certain pathogen-derived enzymes activate plant immunity, BPH-derived CAZymes may have the potential to trigger a defense response in rice plants. This approach could lead to a novel and sustainable strategy for pest management, where CAZymes are used to ‘prime’ the plant’s immune system, preparing it for insect attacks. However, the specific CAZymes involved, as well as their efficiency in triggering immunity, need to be experimentally validated. Further research will be required to explore whether BPH CAZymes can effectively mimic the plant’s response to insect feeding and enhance its resistance.

### 4.2 The number of cell wall enzymes in CAZymes of herbivorous insects is related to their virulence level

During the millions of years of plant and insect co-evolution, herbivorous insects have developed diverse feeding strategies to retrieve nutrients from host plants. Because plants are sessile and cannot escape continuous attack by pests, plants have evolved various defense strategies to combat herbivory. Meanwhile Herbivorous insects produce a large number of impressive PCWDEs (plant cell wall degrading enzymes) to break down plant cell walls and consume their nutrients for development.

It seems that the ongoing tussle between plants and their herbivorous adversaries has spurred an evolutionary arms race, leading to the emergence of a plethora of cell wall-degrading enzyme, as we’ve previously touched upon. Delving into the CAZyme sequences of the brown planthopper, we’ve uncovered a considerable arsenal of enzymes that are adept at dismantling the complex structures of both plant and fungal cell walls. This includes a range of enzymes capable of breaking down cellulose, xylans, glucans, pectin, and chitin, which are the very building blocks of these cell walls (Vorwerk et al., 2004). In particular, we’ve identified certain classes of CBMs, GHs, and GTs that stand out in their ability to degrade plant cell walls. Notably, CBM14, GH1, GH5, GH9, and GH13 have been pinpointed as the key players in this enzymatic degradation process.

These enzymes are part of the CAZyme families, and they likely contribute to breaking down major plant cell wall components, such as cellulose and hemicellulose, which are critical for nutrient acquisition from rice plants. CBM14 is hypothesized to play a role in the binding of cellulose and hemicellulose, facilitating the enzymatic degradation of plant cell walls by anchoring other enzymes to the substrate. While this has been suggested for other insect species, its exact role in BPH feeding on rice plants remains to be experimentally verified (Trinca et al., 2023). GH1 enzymes are commonly involved in the breakdown of cellulose and hemicellulose, helping to convert these polysaccharides into simpler sugars for BPH’s nutrition. Although GH1 enzymes are well-known in herbivorous insects, their specific involvement in BPH feeding on rice requires further validation to confirm this hypothesis (Marana, 2006). GH5 enzymes, specifically endo-β-1,4-glucanases, are typically associated with breaking down the cellulose network in plant cell walls, making it easier for BPH to access underlying plant tissues. The broad substrate specificity of GH5 enzymes suggests they are likely involved in BPH’s feeding on rice, but experimental studies are needed to confirm this in the context of rice plants (Aspeborg et al., 2012). GH9 enzymes, another class of endo-β-1,4-glucanases, have been identified in a variety of insect species and are implicated in cellulose breakdown. In the case of BPH, these enzymes likely contribute to the degradation of tough rice plant cell walls, but direct experimental evidence of their role in BPH feeding is still needed (Watanabe and Tokuda, 2010). GH13, which includes α-amylases, is potentially important for the breakdown of starch and other carbohydrates in the rice plant. While GH13 enzymes are known to play roles in carbohydrate digestion in other herbivorous insects, their function in BPH feeding on rice plants requires experimental confirmation (Trinca et al., 2023).

These enzymes, if confirmed to play a role in BPH’s feeding strategy, would work together to break down the plant cell wall and facilitate nutrient extraction. This process is essential for BPH’s survival and reproduction. The diversity and specificity of these CAZymes in BPH suggest that they are adapted to feeding on rice plants and overcoming the plant’s structural and chemical defenses. However, while these roles are supported by findings from other insect species, experimental validation is needed to confirm their exact functions in BPH-rice interactions.

When it comes to the breakdown of cellulose, the process is typically orchestrated by a trio of glycoside hydrolases: endoglucanases, exoglucanases, and β-glucosidases, as referenced in (Calderón-Cortés et al., 2012; Tokuda et al., 2007; Watanabe and Tokuda, 2010). Our analysis has revealed several cellulase genes within the brown planthopper that could be instrumental in the decomposition of cellulosic biomass. These include GH5, GH9, GH13, and GH31, each potentially playing a distinct role in the cellulose degradation pathway. Several cellulase genes from the BPH, including GH5, GH9, GH13, and GH31, have potential roles in breaking down cellulose biomass. Cellulase activity has been reported in insects like termites and cockroaches, and similar activities are found across various insect orders (Calderón-Cortés et al., 2010; Watanabe and Tokuda, 2010).

In addition to cellulases, proteins like GH16 and GH47 play a role in hemicellulose degradation, with GH16 associated with xyloglucan endotransglucosylase, important for modifying the plant cell wall (Doi and Kosugi, 2004). Pathogenic fungi, such as *Ustilago maydis*, employ enzymes like β-1,3-glucanases to remodel plant cell walls and escape immune responses. Insects, including BPH, secrete a variety of PCWDEs to degrade plant defenses, aiding in feeding and colonization (Azizi et al., 2014; Kawasaki et al., 2017).

The presence of these enzymes enhances the virulence of herbivores, as they help bypass plant defenses and promote nutrient acquisition from host plants (Hao et al., 2008; Ji et al., 2017; Kobayashi, 2016). CAZymes such as the β-1,4-glucanase NlEG1 in BPH facilitate the degradation of rice cellulose, allowing the BPH to access phloem and enhance its feeding efficiency (Ji et al., 2017).

An interesting aspect of the BPH feeding process is the ability of CAZymes to alter plant cell wall structures, weakening the plant’s defense. Previous studies have shown that increasing the deposition of certain cell wall components like lignin and callose in response to herbivory enhances plant defenses (Hao et al., 2008). In theory, if plant cell walls could be structurally reinforced, it may deter BPH feeding. Enhancing cell wall integrity through genetic modifications or exogenous treatments (e.g., boosting lignin or cellulose deposition) could reduce the accessibility of plant nutrients and decrease BPH infestation. However, the exact mechanisms behind how these modifications influence BPH feeding and their feasibility in real-world applications remain poorly understood. Future studies should focus on the potential trade-offs of enhancing cell wall structure, such as impacts on plant growth and nutrient transport, alongside the effects on herbivore resistance.

### 4.3 The relationship between BPH CAZyme response and host resistance genes

During co-evolution with pathogens, plants have developed a robust innate distinct layers of defense mechanisms. The first layer, pattern-triggered immunity (PTI), relies on plasma membrane-bound pattern recognition receptors (PRRs) that detect pathogen-associated molecular patterns (PAMPs), triggering an immune response. The second layer, effector-triggered immunity (ETI), is activated when plant receptors recognize pathogen effectors, particularly nucleotide-binding leucine-rich repeat receptors (NLRs). Increasing evidence indicates that these two branches of plant immunity, which are critical for defending against pathogens, also play a role in insect resistance (Dodds and Rathjen, 2010; Saile et al., 2020; Wang et al., 2019).

The plant cell wall serves as a physical barrier during immune responses, playing a key role in immune recognition. When insects feed on plants, the plant perceives the attack and initiates defense responses, which include the deposition of callose and lignin in the cell wall. This process strengthens the wall, enhancing its barrier function and limiting insect access to essential nutrients (Wu et al., 2022).

PTI initiates upon the recognition of PAMP or microbe-associated (MAMPs) by receptor kinases such as *Bph3*, whereas ETI is mediated by intracellular proteins encoded by *R* genes such as *Bph6*, *Bph9*, and *Bph14* (Dogimont et al., 2014; Kellner et al., 2014; Wang et al., 2022; Yan et al., 2023). These immune responses trigger structural changes, including callose deposition and cell wall thickening, which hinder BPH feeding and reproduction. For example, *Bph30* upregulates cellulose and hemicellulose biosynthesis in sclerenchyma cells, significantly thickening and hardening the cell wall (Shi et al., 2021).

To counteract these plant defenses, herbivorous insects, including BPH, produce a diverse array of CAZyme effector proteins. These include pectinases, cellulases, amylases, enzymes that hydrolyze sucrose, and oxidases, which serve multiple functions. BPH, for instance, secretes CAZyme effectors like NlEG1, an enzyme that degrades plant cell wall cellulose, thereby weakening the plant’s defense mechanisms (Ji et al., 2017). Additionally, these enzymes alter the structure of the plant cell wall, particularly targeting components like lignin, hemicellulose, and pectin (Antony et al., 2017). By modifying these structural components, the enzymes enhance the permeability of the cell wall, facilitating better nutrient extraction by the insect. Moreover, these enzymes also suppress the transmission of defense-related signaling molecules, such as salicylic acid (SA), jasmonic acid (JA), and jasmonoyl-isoleucine (JA-Ile). This suppression prevents the plant from activating its defense responses, including the synthesis of anti-insect substances like phenolic glycosides, flavonoids, and other phytoalexins (Fu et al., 2020; Jiang et al., 2024).

In summary, the CAZymes secreted by BPH play a critical role in suppressing plant immune responses, both by modifying the plant cell wall and by interfering with the transmission of immune signals, ultimately allowing BPH to overcome plant defenses and feed more efficiently. This intricate relationship between plant immunity and insect CAZyme activity underscores the adaptive strategies evolved by BPH to optimize feeding and colonization on rice plants.

Our results highlight distinct patterns of gene expression in BPH across different resistant rice varieties, including genes that change consistently across multiple varieties and those that vary specifically with individual varieties. Three glycosyltransferase-related genes (*MSTRG.25023.4*, *MSTRG.36466.1*, and *MSTRG.5824.1*) exhibited consistent upregulation in multiple resistant rice varieties, including RHT and IR56, compared to TN1. This suggests these genes may be broadly associated with counteracting resistance conferred by common mechanisms such as those governed by Bph3. Similarly, chitinase-related genes (*MSTRG.7223.4*, *rna27461*, and *rna38279*) were consistently downregulated across several resistant varieties, indicating a shared suppression of these enzymatic activities by plant defenses, potentially through enhanced ETI pathways. These consistent changes likely reflect core BPH strategies for host adaptation or shared host plant defense responses. In contrast, other CAZyme-related genes exhibited variety-specific changes. For example, *MSTRG.15.4* was significantly upregulated only in IR42, indicating a unique response to the specific resistance pathways in this variety. *MSTRG.5824.1* and *rna29850* were significantly upregulated in IR56, reflecting adaptation to IR56-specific resistance mechanisms. In RHT rice, unique upregulation of *MSTRG.25023.4* and *MSTRG.36466.1* suggests a tailored response to RHT-specific resistance traits, which include enhanced recognition by *Bph3*. These variety-specific responses underline the versatility of BPH in modulating its enzymatic toolkit to overcome distinct resistance strategies deployed by different rice varieties.

### 4.4 Relationship between subcellular localization of candidate genes and brown planthopper infestation

Research indicates that plant immunity against herbivorous insects relies on the innate capabilities of individual plant cells. Plant cells are equipped with a variety of cell surface and intracellular immune receptors capable of perceiving “danger” signals, integrating this information, and activating defense mechanisms against potential herbivore attacks, forming a robust defense system against insect threats. The role of the plasma membrane (PM) in this process is well-documented in plant-pathogen interactions, where the PM is critical for the first stage of immune activation (Boller and Felix, 2009; Schulze-Lefert and Panstruga, 2011; Chen et al., 2022; Wang et al., 2022). Recent studies have shown that PM-localized proteins are involved in detecting herbivore-induced signals and activating immune responses in plants (Bonaventure, 2012).

The initial stage of the immune response involves the recognition of microbe- or herbivore-derived molecular patterns, such as MAMPs and DAMPs (damage-associated molecular patterns). These receptors, which include PRRs on the PM, are encoded by various plant genes that allow plants to detect a broad spectrum of danger signals (Wang et al., 2022). The activation of this system not only helps defend against pathogens but also plays a crucial role in herbivory recognition, enabling plants to resist insect attacks (Zipfel et al., 2006; Hogenhout and Bos, 2011).

Given the critical role of the PM in plant defense, we hypothesized that BPH CAZymes, localized to the PM, could interact with the plant’s defense system. To explore this, we conducted subcellular localization analyses of candidate proteins involved in cell wall degradation and found that some of these enzymes are localized at the plasma membrane. This positioning may facilitate the initial interaction between BPH and the plant’s defense system, potentially altering cell wall integrity and mitigating the plant’s immune responses, such as callose deposition and lignin accumulation, which typically serve to fortify the plant’s defenses against herbivores.

It’s quite plausible that these proteins upregulated and are found to be secretory in nature, taking up residence at the plasma membrane are the foot soldiers in the frontline of immune recognition at the plant cell membrane, where they might either trigger or dampen the plant’s defense mechanisms, depending on the needs of the BPH for feeding (Wang et al., 2022). This is akin to a delicate balancing act, where the salivary proteins from insects like *Myzus persicae* have been observed to lower the levels of plant secondary metabolites and hinder the accumulation of callose, as noted in reference (Hao et al., 2008; Jaouannet et al., 2014; Ji et al., 2017).

The very survival of plants hinges on their capacity to recognize the threat posed by herbivores and to mount an immune response accordingly. This involves a cascade of reactions, including the activation of MAPK pathways, fluctuations in calcium ion levels, and the generation of reactive oxygen species (ROS) along with other signaling molecules (Hõrak, 2020; Hu et al., 2011; Ji et al., 2017; Lecourieux et al., 2006). In a countermove, the BPH might be deploying salivary effectors to quell these plant defenses, thereby smoothing the path for feeding. This hypothesis is supported by the observed presence of NlEG1 in the rice phloem, as detailed in reference (Ji et al., 2017).

The interplay between insect-derived exogenous proteins and plant cell membranes is a complex tapestry, woven with threads of immune recognition and evasion. Delving deeper into this interaction promises to unlock the secrets of the molecular mechanisms that govern herbivore-host dynamics, potentially unveiling new strategies for managing the BPH and other agricultural pests that could revolutionize our approach to pest control. It’s a frontier of research that holds the promise of not just understanding, but also manipulating these intricate relationships, turning the tables in favor of our agricultural endeavors.

## 5 Conclusion

In our quest to unravel the intricacies of the BPH prowess as a formidable agricultural pest, we embarked on a comprehensive study to investigate the diversity and functional significance of CAZymes within its genome. Our analysis unveiled a considerable arsenal of 644 CAZyme genes, accounting for a noteworthy 3.23% of the BPH’s entire genetic arsenal. These enzymes, especially those that partake in the degradation of cell walls, are instrumental in the BPH’s capacity to inflict damage upon rice plants by orchestrating the breakdown of plant cell walls during the feeding process. Our subcellular localization analysis lent further credence to the pivotal role of the CAZymes identified in our study, as they were predominantly found to be localized to the plasma membrane. This finding underscores their active involvement in the interaction with plant cell walls, suggesting a direct role in the plant’s defense mechanisms. Moreover, our research shed light on the adaptive prowess of the BPH, as its CAZyme profile is not static but is influenced by changes in the host plant and the developmental stages of the insect itself. We observed significant variations in gene expression patterns during feeding, which is a testament to the dynamic nature of the BPH’s interaction with its host.

## Data Availability

The original contributions presented in the study are included in the article/**Supplementary Material**. Further inquiries can be directed to the corresponding authors.

## Supplementary material

Supplementary Figure 1 A maximum likelihood tree was constructed using protein sequences from the Carbohydrate Binding Module (CBM) family identified in the brown planthopper, for the plant cell wall-degrading enzymes.

Supplementary Figure 2 A maximum likelihood tree was constructed using protein sequences from the glycosyltransferase (GT) family identified in the brown planthopper, for the plant cell wall-degrading enzymes.
