# Supplementary figures and images for: Role of carbohydrate-active enzymes in brown planthopper virulence and adaptability
Source: Front Plant Sci. 2025 Apr 4;16:1554498. doi: 10.3389/fpls.2025.1554498 (PMC12038449; doi:10.3389/fpls.2025.1554498)

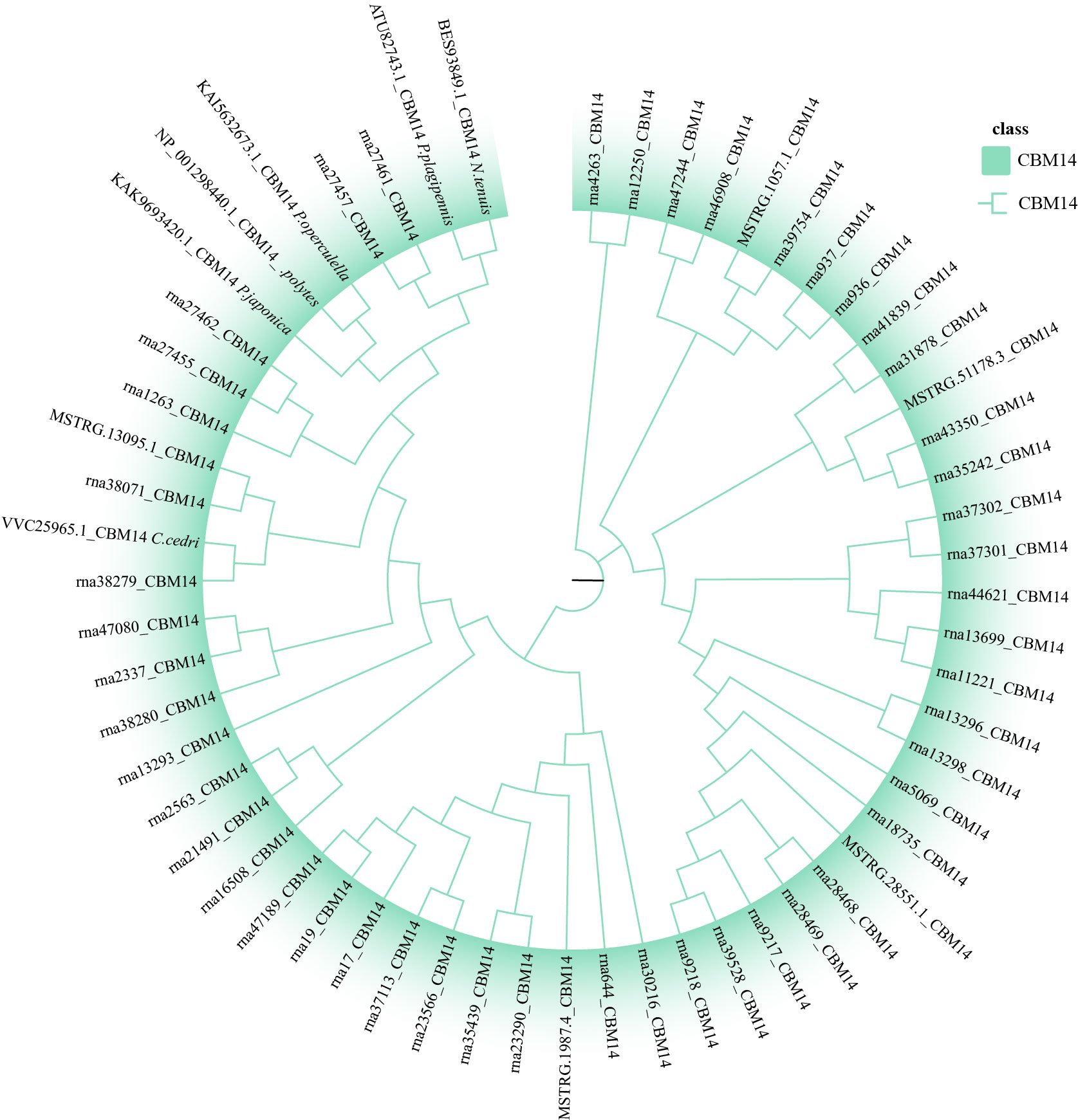

Supplement: Supplementary Figure 1 — A maximum likelihood tree was constructed using protein sequences from the Carbohydrate Binding Module (CBM) family identified in the brown planthopper, for the plant cell wall-degrading enzymes. [file Image1.jpeg]

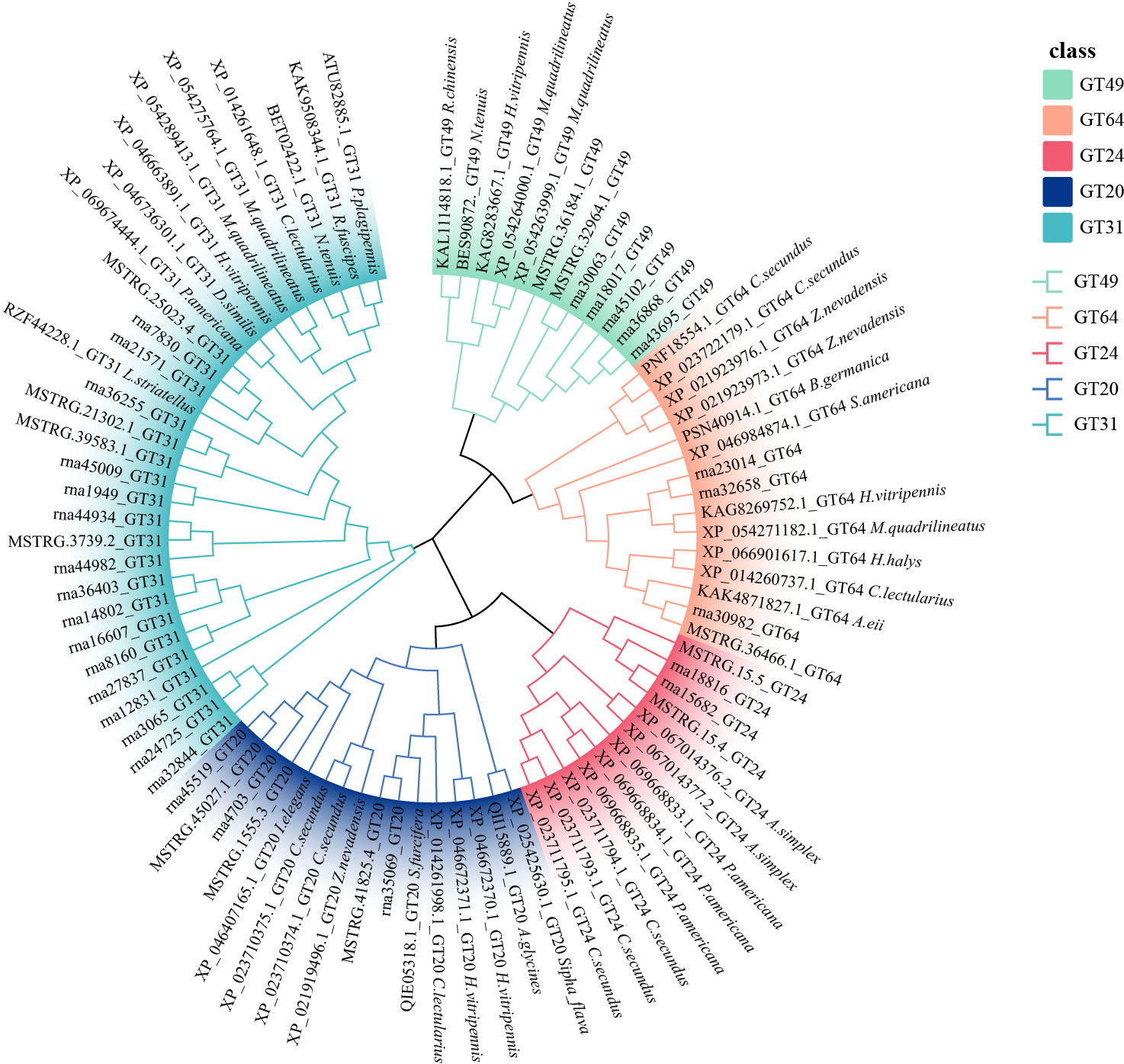

Supplement: Supplementary Figure 2 — A maximum likelihood tree was constructed using protein sequences from the glycosyltransferase (GT) family identified in the brown planthopper, for the plant cell wall-degrading enzymes. [file Image2.jpeg]
